# Helminths: Immunoregulation and Inflammatory Diseases—Which Side Are *Trichinella* spp. and *Toxocara* spp. on?

**DOI:** 10.1155/2013/329438

**Published:** 2013-01-09

**Authors:** Carmen Aranzamendi, Ljiljana Sofronic-Milosavljevic, Elena Pinelli

**Affiliations:** ^1^Centre for Infectious Disease Control Netherlands, National Institute for Public Health and the Environment (RIVM), P.O. Box 1, 3720 BA Bilthoven, The Netherlands; ^2^Institute for the Application of Nuclear Energy (INEP), University of Belgrade, Banatska 31b, 11080 Belgrade, Serbia

## Abstract

Macropathogens, such as multicellular helminths, are considered masters of immunoregulation due to their ability to escape host defense and establish chronic infections. Molecular crosstalk between the host and the parasite starts immediately after their encounter, which influences the course and development of both the innate and adaptive arms of the immune response. Helminths can modulate dendritic cells (DCs) function and induce immunosuppression which is mediated by a regulatory network that includes regulatory T (Treg) cells, regulatory B (Breg) cells, and alternatively activated macrophages (AAMs). In this way, helminths suppress and control both parasite-specific and unrelated immunopathology in the host such as Th1-mediated autoimmune and Th2-mediated allergic diseases. However, certain helminths favour the development or exacerbation of allergic responses. In this paper, the cell types that play an essential role in helminth-induced immunoregulation, the consequences for inflammatory diseases, and the contrasting effects of *Toxocara* and *Trichinella* infection on allergic manifestations are discussed.

## 1. Introduction

Immune responses induced by helminths are predominantly of the Th2 type involving cytokines such as interleukin-3 (IL-3), IL-4, IL-5, IL-9, IL-10, and IL-13. These cytokines mediate immune responses typically characterized by increased levels of circulating IgE antibodies, eosinophils, basophils, and mast cells [1]. During infection, the immune system is exposed to different helminth-derived molecules, including proteins, lipids, and glycoconjugates present either at the surface of the worms or in the excretory-secretory (ES) products [2]. Interaction of helminth-derived molecules with host cells can result in a shift of the immune response, from an inflammatory towards an anti-inflammatory type of response. Helminth-derived molecules can modify dendritic cells (DCs) function and downregulate adaptive immune responses, through the induction of a regulatory network that include regulatory T (Treg) cells, alternatively activated macrophages (AAMs), and regulatory B (Breg) cells. The induced immunosuppresive network, together with cytokines produced by diverse hematopoietic and nonhematopoietic cells as integral part of immunoregulatory pathways, appears to be essential for parasite survival and its effect can be extended to other inflammatory disorders such as allergies and autoimmune diseases [3, 4]. However, the association between helminth infections and allergy does not always have an unequivocal outcome. While certain helminth infections protect against allergic diseases (reviewed in [5]), other helminths can exacerbate this immunopathology (reviewed in [6]). Here, the role of DCs, Treg, and other regulatory cells in helminth-induced immunoregulation, the consequences for inflammatory diseases, and the contrasting effects of *Toxocara *and *Trichinella *infections on allergic manifestations are discussed.

## 2. Dendritic Cells 

DCs are sentinels on alert for possible danger signals to immediately activate local innate immune responses and subsequently, after antigen presentation, initiate the proper adaptive immune responses. Interaction with DCs determines the function and cytokine production of lymphocytes. DCs play therefore an essential role in shaping the immune response and controlling the course of infection [7]. These cells are located throughout the body forming a complex network that allows them to communicate with different populations of lymphocytes. Different DC subsets may have distinct locations, where they acquire antigens to be transported to the draining lymph nodes for T-cell priming [8]. DCs as well as other innate immune cells possess various families of pattern recognition receptors (PRRs) such as Toll-like receptors (TLRs), NOD-like receptors, RIG-like receptors, and the C-type lectin receptors (CLRs) that allow them to recognize a great variety of pathogen-associated molecular patterns (PAMPs). After pathogen recognition via various PRR, DCs produce molecules that induce polarization of different types of responsiveness such as Th1-, Th2-, Th17-, or Treg-related. The response of DCs to pathogens is mediated in large part via TLR, with input from other PRR resulting in changes in gene expression that leads to DCs maturation. Maturation of these cells refers to a transition from a resting state into a more dynamic state in which the cells present antigen in the context of MHC, express costimulatory molecules such as CD40, CD80, and CD86, and secrete a broad spectrum of cytokines and chemokines [9]. TLR-mediated responses are controlled mainly by the MyD88-dependent pathway, which is used by all TLR except TLR3- and by the TIR-domain-containing adapter-inducing interferon-*β*-(TRIF)-dependent pathway, which is used by TLR3 and TLR4 [10]. TLRs have been implicated in the recognition of helminth products by DC. For instance, Lacto-N-fucopentaose III (LNFPIII) produced by trematode *Schistosoma mansoni, *and ES-62, a phosphorylcholine-containing protein secreted by the nematode *Acanthocheilonema viteae*, can condition DCs to induce Th2 responses through TLR4 [11]. Likewise, monoacetylated phosphatidyl serine lipids from schistosomes specifically instruct DCs to preferentially induce IL-10-producing Treg in a TLR2-dependent manner [12]. This was also demonstrated in TLR2-deficient mice that showed a reduced number of CD4+CD25+ Treg cells and immunopathology during schistosomiasis [13]. CLRs on DCs also play an important role in sensing helminth glycans. Studies using schistosomal antigens suggest that helminth glycans may be the conserved molecular pattern that instructs DCs via CLR to drive Th2-polarized responses [14]. Other recent studies demonstrate that host-like glycan antigens expressed by many helminths are recognized by DCs via lectin receptors [2]. Schabussova et al. reported on blood group-like glycans from *T. canis* that bind the lectin DC-SIGN (Dendritic Cell-Specific Intercellular adhesion molecule-3-Grabbing Nonintegrin) [15], which may enable the activation of signal transduction pathways involving Raf-1 and subsequent modulation of DC maturation resulting in skewing towards a Th2 responses [16]. Lewis X antigen, a host-like glycan expressed on the surface of schistosomes in all life stages which is also present in secreted products such as the soluble egg antigens (SEAs), also binds to DC-SIGN [17].

DC maturation is considered to be essential for DCs to induce T-cell responses. It has become clear that DCs responding to helminth products do not mature in the conventional way upon encountering parasitic antigens but acquire a semimatured status and are still capable of inducing T-cell polarization. Several studies support the findings that helminth products fail to directly activate DCs and other studies show that helminth products suppress DC maturation. For instance, SEA suppresses lipopolysaccharide- (LPS-) induced activation of immature murine DCs, as indicated by decreased MHC class II and costimulatory molecules expression in addition to IL-12 production. This resulted in increased LPS-induced production of IL-10 by DC after incubation by SEA [18]. Pretreatment of DCs and macrophages with ES-62 also inhibits their ability to produce IL-12p70 in response to LPS [19]. In another study, a mixture of high molecular weight components from *Ascaris suum* was found to reduce the LPS-induced expression of MHCII, CD80, CD86, and CD40 molecules on mouse CD11c+ DCs and to hampered T-cell proliferative responses *in vitro*. This inhibitory effect was abolished in IL-10-deficient mice [20]. *Fasciola hepatica* tegumental antigen alone did not induce cytokine production or cell surface marker expression on murine DCs; however, it significantly suppressed cytokine production and cell surface marker expression in DCs matured with a range of TLR and non-TLR ligands [21]. *In vitro* studies on the impact of *T. spiralis *excretory/secretory products (TspES) on mice DCs revealed that these parasitic antigens suppress DC maturation induced by LPS derived from different bacteria [22]. In this study, different TLR agonists were used showing that the suppressive effect of TspES on DC maturation is restricted to TLR4. These helminth products were also shown to interfere with the expression of several genes related to the TLR-mediated signal transduction pathways. For rat bone-marrow-derived DC it has been shown that after incubation with TspES these DCs acquire a semimatured status which is reflected in moderate upregulation of CD86, significant upregulation of ICAM1 (Intercellular Adhesion Molecule 1), and no upregulation of MHC II, accompanied by impaired production of IL-12 p70 [23].

## 3. Regulatory T Cells

Treg cells control peripheral immune responses and are likely to play a central role in autoimmune, infectious, and allergic diseases. Three phenotypes of Treg have been described to date, categorized according to their origin, function, and expression of cell surface markers: natural Treg cells (CD4+CD25+Foxp3+) and inducible Treg cells that include the IL-10-producing Tr1 cells and the Foxp3+ T cells induced in the periphery [24]. In spite of the complexity of regulatory cell types, CD4+CD25+Foxp3+ Treg cells are the most prominent population of immunoregulatory cells known so far to be induced during helminth infections [4].

Early studies had already suggested regulatory T-cell activity during chronic helminth infections in humans. Doetze et al. reported that IL-10 and transforming growth factor-*β* (TGF-*β*) production mediated the hyporesponsiveness observed in PBMC from individuals with generalized onchocercosis caused by the filarial nematode *Onchocerca volvulus* [25]. In a study with filariasis patients, lymphedema was associated with a deficiency in the expression of Foxp3, GITR (glucocorticoid-induced tumour-necrosis-factor-receptor-related protein), TGF-*β*, and CTLA-4 (cytotoxic T-lymphocyte antigen 4), known to be expressed by Treg cells [26], while in children infected with intestinal nematodes (*Ascaris lumbricoides* and *Trichuris trichiura*) high levels of IL-10 and TGF-*β* in addition to generalized T-cell hyporesponsiveness were found [27, 28]. Likewise, *Schistosome*-infected individuals in Kenya and Gabon had higher CD4+CD25+ and CD4+CD25+ Foxp3+ T-cell levels compared with uninfected individuals [29]. One of the studies providing evidence on the suppressive effect of Treg cells from helminth-infected individual is the one reported by Wammes et al. [30]. In this study, carried out in Indonesia, Treg cells from geohelminth-infected individuals were more effective at suppressing proliferation and IFN-*γ* production by effector T cells in response to malaria antigens and BCG than Treg cells from healthy individuals. A filarial parasite of humans, *Brugia malayi*, was found to secrete TGH-2 (transforming growth factor homologue-2), a homologue of host TGF-*β* [31]. Since the recombinant TGH-2 can bind to the mammalian TGF-*β* receptor, it has been suggested that it can promote the generation of regulatory T cells, as it has been shown for mammalian TGF-*β*. In another study a significant increased expression of Foxp3 and regulatory effector molecules such as TGF-*β*, CTLA-4, PD-1 (programmed death 1) and ICOS (inducible costimulatory molecule) was found in filaria-infected compared to uninfected individuals in response to live infective-stage larvae or microfilariae of *Brugia malayi *[32].

Various studies on the role of Treg cells in helminth infections have used animal models. In mice, CD25+ Treg cells were shown to restrain the pathology to helminth eggs during schistosome infection [13] and to *Trichuris muris* in the gut [33]. Moreover, depletion of CD25+ Treg cells with combined antibodies to CD25 and GITR resulted in enhanced immunity to filarial nematode *Litomosoides sigmodontis* in mice [34]. Generation of Treg cells with elevated expression of Foxp3 during helminth infection has also been demonstrated. For instance, infection of BALB/c mice with *Brugia pahangi* third-stage larvae (L3) resulted in expansion of a population of CD4+CD25+ T cells which was highly enriched in Foxp3 and IL-10 gene expression [35]. Induction of Treg cells was demonstrated to be necessary to establish a chronic *L. sigmodontis* infection since depletion of Treg cells in susceptible mice prior to infection enhanced parasitic killing and cleared the infection [36]. In chronic infection with the gastrointestinal helminth *Heligmosomoides polygyrus*, it was established that levels of Foxp3 expression within the CD4+ T-cell population of mice mesenteric lymph nodes were significantly increased and that purified CD4+CD25+ Treg cells possess suppressive activity *in vitro* [37, 38]. 

The effect of TspES on T-cell activation *in vitro* was investigated using splenocytes derived from ovalbumin- (OVA)-TCR transgenic D011.10 mice that were incubated with TspES-pulsed DC+OVA. Results indicate that the presence of TspES resulted in expansion of CD4+CD25+ T cells that express high levels of Foxp3+. These Treg cells were shown to have suppressive activity and to produce TGF-*β*. Together these results indicate that *T. spiralis* secretion products can induce expansion of functional Treg cells *in vitro* [22].

In a rat model, the infection with *T. spiralis* is accompanied with the increase proportion of Foxp3+ Treg cells [23]. *In vitro* studies showed that DCs stimulated with TspES caused strong Th2 polarization, accompanied by elevated production of the regulatory cytokines IL-10 and TGF-*β* [23]. However, unlike the mouse model described previously, conditioned rat DCs generated no increase in the proportion of CD4+CD25+Foxp3+ T cells. * In vivo* T-cell priming with TspES stimulated DCs resulted in mixed Th1/Th2 cytokine response, with the dominance of the Th2 type and elevated levels of regulatory cytokines. Significant increase in the proportion of CD4+CD25+Foxp3+ cells was found in spleen cells of recipients that received TspES stimulated DCs compared to the control value obtained from rats that received DCs cultivated in medium only.

## 4. Other Regulatory Cells

Helminth infections may also lead to expansion of immunoregulatory cells other than Treg cells, including alternatively activated macrophages (AAMs) and regulatory B cells. Signals encountered during migration by developing macrophages determine their function at sites of inflammation or infection. Among these signals, cytokines are responsible for the development of highly divergent macrophage phenotypes: classically activated and AAMs [39]. Prieto-Lafuente et al. reported that the homologues of the mammalian cytokine macrophage migration inhibitory factor (MIF) expressed by *Brugia malayi* synergized with IL-4 to induce the development of suppressive AAMs *in vitro* [40]. One pathway for this effect may be through the MIF-mediated induction of IL-4R expression on macrophages, amplifying in this way the potency of IL-4 itself. Thus, in a Th2 environment, MIF may prevent the classical activation of macrophages. The suppressive effect of AAMs on the immune response is most likely dependent on the expression of arginase-1 (Arg-1) as indicated by studies in which mice macrophages lacking Arg-1 failed to suppress Th2 responses (reviewed in [41]). 

B cells possess a variety of immune functions, including production of antibodies, presentation of antigens, and production of cytokines. IL-10-producing regulatory B cells have great potential to regulate T-cell-mediated inflammatory responses [5] and to downmodulate experimental autoimmune encephalomyelitis, collagen-induced arthritis, and inflammatory bowel disease [42]. In addition, in mouse models of chronic parasitic inflammation, such as chronic schistosomiasis, IL-10-producing B cells were also reported to be associated with protection against anaphylaxis [43]. Moreover, *H. polygyrus*-infected mice induced regulatory B cells that can downmodulate both allergy and autoimmunity in an IL-10 independent manner [44].

## 5. Helminth Infections and Inflammatory Diseases

The helminth-induced immunosuppresive network may not only be beneficial for the parasite, but it can also have beneficial outcomes for the host, reducing allergic and autoimmune diseases [41, 45]. Epidemiological, cross-sectional studies support an inverse correlation between allergic diseases and helminth infection [46, 47] including infections by nematode species like *A. lumbricoides* and *Necator americanus* [48]. An increased skin reactivity to house dust mites was found after antihelminthic treatment against infection with *A. lumbricoides and T. trichiura* [49]. Studies performed in animal model system have confirmed that helminth infection can protect against allergic disease and in particular lung-associated inflammation. For instance, *S. mansoni*-infected BALB/c mice were protected against OVA-induced experimental allergic airway inflammation (EAAI) as indicated by reduction of eosinophils in BAL, Th2 cytokine production, OVA-specific IgE levels and reduction of the number of inflammatory cells in lungs. Here, induction of CD4+CD25+Foxp3+ regulatory T cells was independent of IL-10 [50]. Dittrich et al. found that chronic infection with the filarial parasite *Litomosoides sigmodontis *suppressed all pathological features of the OVA-induced EAAI model [51]. Additionally, these authors observed significantly increased numbers of Treg cells in spleen and mediastinal lymph nodes in infected OVA-treated mice compared to OVA-controls animals. Suppression of EAAI during the course of *H. polygyrus* infection was shown to involve the induction of Treg cells [52]. Infection with the same parasite resulted also in the inhibition of allergic response to peanut extract [53]. 

Several epidemiological studies have investigated the protective effect of parasitic infections in different autoimmune diseases like multiple sclerosis and type 1 diabetes [54]. Studies indicate that persons infected with chronic parasitic worm infections have lower rates of inflammatory bowel disease (IBD) than persons without these infections [55]. Experiments carried out using animal models of human autoimmune diseases have shown that parasites can interfere with autoimmunity. *Schistosoma mansoni* infection has been shown to protect from type 1 diabetes [56] and reduces the severity of EAE [57] while infection with *H. polygyrus* suppresses the experimental colitis [58]. Infection with *L. sigmodontis* prevented diabetes in NOD mice. In this study, protection was associated with increased Th2 responses and Treg cell numbers [59].

The immunomodulatory effect of helminth-derived products has been extensively studied.  Table 1 provides an overview of different helminthic antigens with immunoregulatory properties. Findings regarding the use of parasite antigens to suppress experimental inflammatory diseases are summarized in Table 2.

Although the majority of data suggest that infection with helminths is associated with a suppression of allergic and autoimmune responses, some examples provide the opposite view. Epidemiological studies indicate that infection with *Ascaris *spp, *Toxocara *spp, *Fasciola hepatica*, hookworms, or *Enterobius vermicularis* has no protective effects or even enhanced allergic responses (reviewed in [95]).

There are also experimental studies that show that infection with some helminths have a positive association with allergy. A study using a murine model has shown that *T. canis* infection results in exacerbation of EAAI [96]. Other animal experiments provided evidence that parasites like *Nippostrongylus brasiliensis *[97] and* B. malayi* [98] could also induce or exacerbate allergic responses. Exacerbation of anaphylaxis has been shown to occur during *T. spiralis* infection [99]. The links between infections and autoimmunity are complex and there is scarce evidence on the induction or exacerbation of autoimmune responses by helminths [100].

## 6. Contrasting Effect of *Toxocara* and *Trichinella* Infections on Inflammatory Diseases

*Toxocara canis *and *Toxocara cati* are roundworms of dogs and cats, respectively, that can also infect humans worldwide. After ingestion of the infectious *Toxocara* eggs, the larvae migrate to the intestine, liver, and lungs. While in dogs and cats under the age of 6 months, the larva migrate back to the intestine; in humans migration continues to other organs where they can persist for many years [101]. *Toxocara* infection results in the induction of Th2 cells that make cytokines such as IL-4, IL-5, and IL-13, which induce responses to the parasite such as increased IgE levels and eosinophilia (reviewed in [6]). *Trichinella spiralis* is also a roundworm that infects different mammals including humans and mice. After ingestion of *Trichinella* infected meat, the larvae migrate to the intestine and matures to the adult stage, the parasites mate, and finally the newborn larvae (NBL) migrate to striated muscle cells where they become encysted. Infection with *T. spiralis* is characterized by the induction of a Th1 type of response at the beginning of the intestinal phase. When the NBL disseminate, a dominant Th2 type of response develops which is essential for parasite expulsion [102]. Ingestion of both *Toxocara* spp. and *Trichinella* spp. commonly results in chronic infections. Interestingly these helminths have a contrasting effect on inflammatory diseases, while infections with *Trichinella *spp. can suppress (reviewed in [103]) *Toxocara* spp. exacerbate inflammatory diseases [6]. Studies using animal models for human autoimmune and allergic diseases indicate that *Trichinella* infection ameliorates these immune disorders (Table 3). Khan et al. showed that *T. spiralis* infection reduces the severity of dinitrobenzenesulphonic-acid- (DNBS-) induced colitis in C57BL/6 mice [88]. Motomura et al. demonstrated that in addition to the protection exerted by the actual infection, rectal submucosal administration of *T. spiralis* crude muscle larvae antigen can also protect [78]. *T. spiralis *infection also ameliorated autoimmune diabetes in NOD mice [89] and modulated severity of the disease in the experimental model of multiple sclerosis (MS), namely, experimental autoimmune encephalomyelitis (EAE) in Dark Agouti rats in a dose-dependent manner [90]. In this study severity of EAE as judged by cumulative disease index, maximal clinical score, duration of illness, and the number of mononuclear cells infiltrating the spinal cord in *T. spiralis* infected animals were all reduced in comparison to the uninfected EAE-induced group. In a following study, these authors reported that alleviation of the disease in infected-EAE rats coincided with reduced IFN-*γ* and IL-17 production and increased IL-4, IL-10, and TGF-*β* production. They suggested that mechanisms underlying the observed beneficial effect include Th2 and regulatory responses provoked by the parasite. Transfer of T-cell-enriched spleen cells from *T. spiralis*-infected rats that contained a higher proportion of CD4+CD25+Foxp3+ regulatory T cells into rats in which EAE was induced caused amelioration of EAE, which indirectly points to the role of Treg in restraining inflammatory conditions [91]. Boles et al. have shown with another *Trichinella* species, namely, with *Trichinella pseudospiralis*, that infection results in suppression of MS in the rat [93]. These authors used this model to compare the anti-inflammatory effects of the intestinal and late migratory phases of *T. pseudospiralis *infection on development of myelin-basic-protein- (MBP-) induced MS-like debilitation. Findings from this study indicate that the late migratory phase of infection which occurred during the peak of MBP-induced debilitation significantly improved performance scores in mobility, coordination, and strength. Wu et al. also reported on amelioration of clinical severity and delayed onset of EAE after *T. pseudospiralis *infection. This effect was associated with suppression of Th17 and Th1 responses induced by infection [94]. *Trichinella pseudospiralis* is markedly different from *T. spiralis* in that it is smaller in size and that the muscle stage larvae are not surrounded by a capsule [104]. Whether the mechanisms involved in immunosuppression varies depending on *Trichinella* species remains to be investigated.

**Figure 1:**
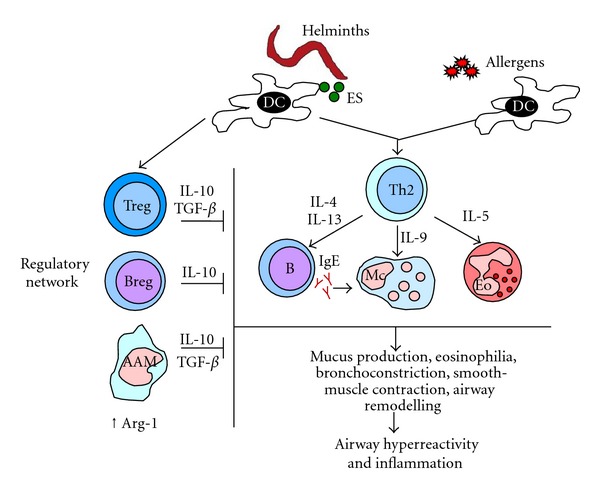
Mechanisms involved in immunosuppression induced by helminths and its effect on allergic responses. Helminths can modulate dendritic cells (DCs) function and induce regulatory T (Treg) cells. Other cells from the regulatory network include regulatory B (Breg) cells and alternatively activated macrophages (AAMs). These cells create an immunosuppressive (⊣) environment in which increased expression of arginase 1 (Arg 1) in AAMs and the production of the cytokines IL-10 and TGF-*β* play an essential role in reducing allergic effector mechanisms.

Infection with *T. spiralis* can also ameliorate EAAI [92]. In this study, the concentrations of IL-10 and TGF-*β* were significantly increased and the recruitment of Treg into draining lymph nodes was elevated as the result of *T. spiralis* infection. This protective effect has been recently shown to occur during acute as well as chronic phases of *Trichinella *infection [105]. Protection against EAAI to OVA was stronger during the chronic phase of infection and associated with increased numbers of splenic CD4+CD25+Foxp3+ Treg cells with suppressive activity. Adoptive transfer of CD4+ T cells from chronically infected mice with elevated numbers of Treg cells in the spleen induced partial protection against EAAI [105]. The possible mechanisms by which helminths or their products could inhibit allergic responses are depicted in Figure 1.

 In contrast to the suppressive effect of *Trichinella* infections on allergic diseases experimental as well as epidemiological studies indicate that *Toxocara* infections are risk factors for allergies, including allergic asthma [6].

Studies using murine models for toxocariosis indicate that infection with *T. canis* leads to persistent pulmonary inflammation, eosinophilia, increase levels of circulating IgE, airway hyperreactivity, and production of Th-2 type cytokines. Pulmonary inflammation has been shown to develop as soon as 48 hours after infection, it occurs in a dose-dependent manner, and it can persist up to 2 or 3 months. Eosinophil counts also increase in the bronchoalveolar lavage (BAL) of *Toxocara*-infected mice [106, 107]. Relative quantification of cytokine expression in lungs of mice infected with different *T. canis* doses showed that while a proportional increased expression of the IL-4, IL-5, and IL-10 transcripts was observed, the expression of IFN-*γ* was not different from that of uninfected controls [107]. Results from this study indicate that infection of mice with *T. canis* results in a dominant Th2 type of immune response, independent of the inoculum size [108]. In addition, infection of BALB/c mice with 1,000 *T. canis* embryonated eggs results in hyperreactivity of the airways that persisted up to 30 days p.i. Evaluation of parasite burden revealed that few *T. canis* larvae were still present in the lungs of infected mice at 60 days p.i. which could explain the persistent pulmonary inflammation observed in these mice [107]. 

Common features between allergic asthma and toxocariosis are the induction of a Th2-cell mediated immune response including the production of high levels of IgE, and eosinophilia. In addition, infection with *Toxocara *spp. shares common clinical features with allergic asthma such as inflammation of the airways accompanied with wheezing, coughs, mucus hypersecretion, and bronchial hyperreactivity. In order to study the effect of *Toxocara* infection on allergic manifestations two murine models were combined, namely, the murine model for toxocariosis described above and a murine model for allergic airway inflammation. For this study BALB/c mice were infected with 500 embryonated *T. canis* eggs and exposed to OVA sensitization followed by OVA-challenge. Results indicate that infection with *T. canis* in combination with OVA treatment led to exacerbation of pulmonary inflammation; eosinophilia; airway hyperresponsiveness; increase of OVA specific and total IgE; increased expression of IL-4 compared to mice that were only *T. canis* infected or OVA treated. The observed exacerbation of EAAI was independent of the timing of infection in relation to allergen exposure. In conclusion, infection with *T. canis* leads to exacerbation of EAAI [96]. 

Several factors may influence the differential effect of helminth infections on allergic diseases [6]. One of these factors is whether the host is definitive or accidental. The normal or definitive hosts for *T. canis *are dogs whereas humans are accidental hosts for this parasite. In an accidental host the parasite does not usually develop to the adult stage and in case of *Toxocara* spp. the continuous migration of the larvae through different organs including the lungs can cause more damage comparing to what happens in a definitive host where migration is transitory. *T. spiralis* can infect many different mammals including mice and humans in which the parasite completes its life cycle. The infected mammal is therefore a definitive host for this helminth. And although *T. spiralis* pass through the lung microvascular system on its way to the skeletal muscle, it is a rapid process in which the larvae are usually not trapped in the lungs [109]. It is likely that there are differences between parasites of humans such as *T. spiralis* that have evolved with their host and have developed strategies to survive without causing much damage compared to parasites such as *Toxocara *spp. for which humans are accidental host [6, 110]. 

In conclusion, helminths induce an anti-inflammatory response, which could ameliorate inflammatory diseases; however, this is not a universal property of all helminths and different factors such as the helminth species, and whether the host is definitive or accidental, the parasite load and acute versus chronic infections may all influence the overall effect of helminth infections on inflammatory diseases. Identification of the helminth molecules that induce immunosuppression and elucidation of the mechanisms involved is essential for the development of alternative strategies for prevention and/or treatment of inflammatory diseases.

## Figures and Tables

**Table 1:** Helminth-derived antigens with immunoregulatory properties.

Helminth	Antigen	Immunoregulatory mechanism	References
*Schistosoma mansoni *	LNFPIII (lacto-*N*-fucopentaoseIII)/SEA (soluble egg antigen)	Interact with TLR4 to produce Th2 polarizing DCs	[60]

*Schistosoma mansoni *	Schistosome lysophosphatidylserine	Interact with TLR2 to induce Treg polarizing DCs	[61]

*Acanthocheilonema viteae *	ES-62	Exert immunomodulatory effects on macrophages and DCs by a TLR4-dependent mechanism with consequent Th2 polarisation	[62–64]

*Nippostrongylus brasiliensis *	Excretory-secretory antigen (NES)	Potently induce Th2 type of response via DC	[65]

*Brugia malayi* adult	Cystatins (cysteine protease inhibitors) CIP-2	Interfere with antigen processing in human cells and inhibits B cells	[66, 67]
	Cytokine homologue MIF-1/2	Alternatively activate macrophages	[68]

*Brugia malayi* microfilariae	Serpins (serine protease inhibitors) SPN-2	Block neutrophil protease and promote Th1 type of response	[69]

*Brugia malayi* L3 larvae	ALT-1/2 proteins	Inhibit macrophage resistance and present good filarial vaccine candidate	[70]

*Toxocara canis *	TES32—C type lectin (CTL)	Inhibit TLR responses on DC and compete with host lectins for ligands, thereby blocking host immunity	[71, 72]

*Heligmosomoides polygyrus *	Excretory-secretory antigen (HES)	Induce regulatory T cells through TGF-*β*R	[37]

*Teladorsagia circumcincta *	Excretory-secretory antigen	Induce generation of Foxp3+ regulatory T cells through TGF-*β* mimicking effect	[73]

*Trichinella spiralis *	Adult excretory-secretory antigen (AdES); newborn larvae antigen (NBL); crude muscle larvae antigen (MLCr)	All antigens from different life stages induce polarization towards mixed Th1/Th2 with predominance of Th2 response, via semimatured DC	[74]
	Excretory secretory muscle larvae antigen	Induce mixed Th1/Th2 response with the predominance of Th2 component and elicit regulatory arm of immune response	[75]
	Excretory secretory muscle larvae antigen	Interfere with LPS-induced DC maturation and induce expansion of Foxp3+ regulatory T cells	[22]

*Fasciola hepatica *	Thioredoxin peroxides	Alternatively activated macrophages	[76]

**Table 2:** Suppression of experimental inflammatory diseases by parasite-derived antigens.

Helminth	Antigen	Model	Reference
*Trichinella spiralis *	Soluble antigens of muscle larvae	EAE DNBS-induced colitis	[77] [78]
*Trichuris suis *	Soluble antigens of adult worm	EAE	[77]
*Ancylostoma ceylanicum *	Soluble and excretory-secretory antigens of adult worm	DSS-induced colitis	[79]
*Hymenolepis diminuta *	Soluble antigens of adult worm	DNBS-induced colitis	[80]
*Heligmosomoides polygyrus *	Excretory-secretory antigens (HES) of adult worm	EAAI	[73]
*Ancylostoma caninum *	Excretory-secretory antigens of adult worm	TNBS-induced colitis	[81]
*Acanthocheilonema vitae *	ES-62	Collagen-induced arthritis	[82]
*Schistosoma mansoni *	Soluble antigens of adult worm	TNBS-induced colitis	[81]
	SEA and soluble adult worm antigen	T1D	[83]
	Recombinant proteins (Sm22*·*6, Sm29) and soluble adult worm fraction (PIII)	EAAI	[84]
*Schistosoma japonicum *	SEA	EAAI	[85]
*Nippostrongylus brasiliensis *	Excretory-secretory antigens (NES) of adult worm	EAAI	[86]
*Ascaris suum *	Soluble antigens of adult worm	EAAI	[87]

EAE: experimental autoimmune encephalomyelitis; DNBS: dinitrobenzene sulfonic acid; TNBS: trinitrobenzene sulfonic acid; DSS: dextran sodium sulfate; T1D: type 1 diabetes; EAAI: experimental allergic airway inflammation.

**Table 3:** Experimental models of Th2-mediated inflammatory diseases successfully treated by *Trichinella* infection or administration of *Trichinella* antigens.

*Trichinella* spp. or their products	Experimental disease model	Reference
*T. spiralis *	Exp. colitis	[88]
	T1D	[89]
	EAE	[90]
	EAE	[91]
	EAAI	[92]
	EAAI	[22]

*T. pseudospiralis *	EAE	[93]
	EAE	[94]

*T. spiralis* crude muscle larvae antigen	Exp. colitis	[78]
	EAE	[77]

Exp. colitis: experimental colitis; T1D: type 1 diabetes; EAE: experimental autoimmune encephalomyelitis; EAAI: experimental allergic airway inflammation.
